# Key Predictors of Patient Satisfaction and Loyalty in Saudi Healthcare Facilities: A Cross-Sectional Analysis

**DOI:** 10.3390/healthcare12202050

**Published:** 2024-10-16

**Authors:** Abdulaziz M. Alodhialah, Ashwaq A. Almutairi, Mohammed Almutairi

**Affiliations:** 1 Department of Medical Surgical Nursing, College of Nursing, King Saud University, Riyadh 11451, Saudi Arabia; mohalmutairi@ksu.edu.sa; 2 School of Nursing & Midwifery, Monash University, Clayton 3168, Australia; ashwaq.almutairi@monash.edu

**Keywords:** patient satisfaction, patient loyalty, healthcare quality, Saudi Arabia, cross-sectional study

## Abstract

Background: Patient satisfaction and loyalty are essential indicators of healthcare quality, directly impacting patient outcomes and the long-term success of healthcare facilities. Despite the growing importance of patient-centered care in Saudi Arabia, there is limited research exploring the factors that influence patient satisfaction and loyalty, particularly in the Riyadh region. Aim: This study aims to identify the key factors influencing patient satisfaction and loyalty among Saudi patients attending public and private healthcare facilities in the Riyadh region. The study focuses on how healthcare service quality, communication, and demographic factors contribute to patient satisfaction and loyalty. Methods: A cross-sectional study was conducted with a sample of 350 Saudi patients from 10 healthcare facilities in Riyadh. Data were collected using the Patient Satisfaction Questionnaire (PSQ-18) and the Patient Loyalty Questionnaire (PLQ). Descriptive statistics, Pearson correlation, and multiple linear regression were employed to identify predictors of patient satisfaction and loyalty. Results: Significant predictors of patient satisfaction included general satisfaction (β = 0.48, *p* < 0.001), communication (β = 0.35, *p* < 0.001), and the frequency of healthcare visits (β = 0.13, *p* = 0.011). Private healthcare facilities had higher satisfaction (*p* < 0.001) and loyalty scores (*p* < 0.001) compared to public facilities. Patient loyalty was strongly predicted by general satisfaction (β = 0.55, *p* < 0.001) and communication (β = 0.42, *p* < 0.001). Conclusions: Communication quality and patient satisfaction are key drivers of patient loyalty in Saudi healthcare facilities. Private facilities outperform public ones in patient satisfaction and loyalty. These findings emphasize the need for healthcare providers to enhance communication and service quality to foster patient loyalty. Tailored approaches to meet the diverse needs of patients, particularly in terms of education and visit frequency, are crucial for improving healthcare outcomes in Saudi Arabia.

## 1. Introduction

Patient satisfaction and loyalty are critical components of healthcare delivery, directly influencing the overall quality of care, patient retention, and healthcare facility success [1]. Globally, the healthcare industry has shifted from a provider-centered approach to a patient-centered model that prioritizes the needs, preferences, and satisfaction of patients [2,3]. This paradigm shift has been particularly significant in healthcare systems that aim to enhance service quality, optimize patient outcomes, and foster long-term relationships between patients and providers [4]. In Saudi Arabia, the healthcare system has undergone substantial changes in recent years, particularly with the implementation of the Saudi Vision 2030 initiative, which seeks to improve the quality of healthcare services and promote patient satisfaction [5]. However, the factors that contribute to patient satisfaction and loyalty in the Saudi context, particularly in the Riyadh region, remain underexplored [6].

Patient satisfaction refers to the extent to which patients are content with the healthcare services they receive [7]. It is a multidimensional concept that encompasses several aspects of healthcare delivery, including the quality of care, communication between patients and providers, the responsiveness of healthcare staff, and the overall environment of healthcare facilities [8]. Research has consistently shown that satisfied patients are more likely to adhere to treatment plans, experience better health outcomes, and develop a sense of trust and loyalty toward their healthcare providers [9]. Conversely, dissatisfaction with healthcare services can lead to negative health outcomes, reduced trust in the healthcare system, and increased rates of switching between healthcare providers [10,11].

Patient satisfaction is also a key performance indicator for healthcare organizations. It reflects the effectiveness of service delivery, the competence of healthcare staff, and the organization’s ability to meet patient needs [12]. In the Saudi context, where the healthcare system is undergoing rapid transformation, measuring and improving patient satisfaction has become a priority for both public and private healthcare institutions [5,13,14]. However, patient satisfaction in Saudi Arabia is influenced by various cultural, social, and organizational factors, which may differ from those in other regions [15]. Therefore, there is a need for context-specific research that identifies the unique factors affecting patient satisfaction among Saudi patients.

Patient loyalty is closely related to satisfaction but refers specifically to the likelihood that a patient will continue using the same healthcare provider or facility over time [16]. Loyalty is crucial for the long-term success of healthcare organizations, as it is more cost-effective to retain existing patients than to acquire new ones [17]. Moreover, loyal patients are more likely to refer others to the healthcare facility, thereby contributing to the organization’s reputation and patient base [18].

In healthcare settings, patient loyalty is often driven by positive experiences, trust in healthcare providers, and a sense of security in the quality of care [19]. Loyalty also hinges on the perceived value of healthcare services, which includes both the clinical outcomes and the overall patient experience [20]. In Saudi Arabia, where patients have access to both public and private healthcare services, loyalty can be influenced by various factors, including the availability of specialized services, the reputation of healthcare providers, and cultural considerations [15]. However, research on patient loyalty in Saudi healthcare settings is limited, and there is a need to explore the specific factors that contribute to patient loyalty among Saudi patients, particularly in the Riyadh region.

Several factors may influence patient satisfaction and loyalty in Saudi Arabia, including cultural norms, religious values, communication styles, and the organizational structure of healthcare facilities [21]. In Saudi Arabia, the cultural emphasis on privacy and modesty may affect patients’ expectations regarding healthcare services, particularly in terms of gender-segregated care and the availability of female healthcare providers for female patients [22].

While patient satisfaction and loyalty are critical components of healthcare delivery, directly influencing the overall quality of care, patient retention, and healthcare facility success [23,24,25], globally, numerous studies have demonstrated the relationship between patient satisfaction, adherence to treatment, and long-term loyalty, highlighting the importance of communication, service quality, and patient–provider relationships in fostering patient loyalty [26,27,28,29].

In the context of Saudi Arabia, research on patient satisfaction is still emerging. While some studies have focused on clinical outcomes and organizational performance [30], few have explored the specific factors influencing patient satisfaction and loyalty in the region, particularly in the rapidly evolving healthcare landscape under the Saudi Vision 2030 initiative [31]. Existing research has emphasized the importance of provider communication and empathy in shaping patient experiences [32], but there is limited empirical evidence on how these factors influence loyalty, particularly in diverse healthcare settings such as public and private facilities.

This study seeks to fill this gap by examining the key predictors of patient satisfaction and loyalty among Saudi patients attending healthcare facilities in the Riyadh region. By focusing on factors such as the frequency of healthcare visits, the type of facility, and patient demographics, this research aims to provide a more comprehensive understanding of how different aspects of the healthcare experience contribute to patient satisfaction and long-term loyalty. These findings will not only inform healthcare providers in Saudi Arabia but also contribute to the global discourse on improving patient experiences in developing healthcare systems.

**Aim of the Study**

The aim of this study is to evaluate the factors influencing patient satisfaction and loyalty among Saudi patients attending healthcare facilities in the Riyadh region, focusing on aspects such as communication quality, the type of healthcare facility (public vs. private), the frequency of healthcare visits, and the overall healthcare service experience.

**Research Hypothesis**

**H1.**  *Patients attending private healthcare facilities will report higher levels of satisfaction compared to those attending public healthcare facilities*.

**H2.**  *Higher patient satisfaction will be positively associated with higher patient loyalty scores*.

**H3.**  *The frequency of healthcare visits will be positively associated with patient satisfaction, with patients who visit more frequently reporting higher satisfaction scores*.

**H4.**  *Communication quality and interpersonal manner will significantly predict patient loyalty, with higher scores in these domains associated with greater loyalty*.

## 2. Materials and Methods

**Study Design**

This study employed a cross-sectional design to examine the factors influencing patient satisfaction and loyalty among Saudi patients attending healthcare facilities in the Riyadh region. The cross-sectional approach was chosen to gather data at a single point in time, enabling a comprehensive analysis of the factors that contribute to patient satisfaction and loyalty. The study followed the STROBE (Strengthening the Reporting of Observational Studies in Epidemiology) checklist to ensure the accuracy and quality of reporting, adhering to its guidelines for cross-sectional studies [33]

**Setting**

Patients were recruited from a total of 10 healthcare facilities, comprising 5 public and 5 private facilities located within the Riyadh region. The selection of facilities ensured a balance between urban and rural settings, with 3 urban and 2 rural facilities in each category (public and private). These healthcare facilities provided a variety of medical services, including primary care, outpatient specialty services, and emergency care. The specialties covered ranged from general medicine to more specialized services such as cardiology, orthopedics, and internal medicine, allowing the study to capture a broad spectrum of patient experiences across different healthcare needs.

**Sample and Sampling**

The study included a sample of 350 Saudi patients who visited healthcare facilities in the Riyadh region. To ensure that the sample was representative of the diverse population in Riyadh, patients were recruited from both public and private healthcare facilities, including hospitals, outpatient clinics, and primary care centers. This approach enabled the study to capture a broad spectrum of healthcare experiences, from routine check-ups to specialized care.

**Inclusion Criteria:**

1. Patients aged 18 to 75 years.
2. Patients who visited public or private healthcare facilities in the Riyadh region at least once within the last 12 months.
3. Patients with chronic diseases, such as diabetes, hypertension, or cardiovascular disease, who require ongoing care and follow-up.
4. Patients with mild-to-moderate acute conditions (e.g., respiratory infections and skin conditions) who had been treated in primary or secondary care facilities.
5. Patients with stable mental health conditions who were able to provide informed consent.

**Exclusion Criteria:**

1. Patients younger than 18 years or older than 75 years.
2. Patients with severe cognitive impairments (e.g., advanced dementia) or other mental health conditions that would prevent them from providing informed consent or reliably responding to survey questions.
3. Patients undergoing emergency treatment or those in critical care at the time of the study.
4. Patients with rare or complex diseases that require highly specialized care, as these patients might experience different satisfaction and loyalty factors.
5. Patients who had visited the healthcare facility for one-time procedures (e.g., vaccinations or routine screenings) and did not have ongoing healthcare needs.

**Sampling Technique**

A **convenience sampling** method was employed for participant recruitment. This non-probability sampling technique allowed the researchers to approach and recruit patients who were readily available and willing to participate during their healthcare visits. Although convenience sampling has limitations in terms of generalizability, it was considered appropriate for this study given the logistical challenges of recruiting a large number of participants across multiple healthcare facilities in a limited time frame.

**Recruitment Process**

Participants were recruited from the outpatient waiting areas of the selected healthcare facilities. The research team collaborated with healthcare administrators and staff to identify appropriate times and locations for data collection without disrupting the usual flow of patient care. Recruitment was conducted at various times of the day and on different days of the week to ensure a diverse sample of participants with varying schedules and healthcare needs.

Once patients were identified as eligible, they were approached by a member of the research team, who provided them with detailed information about the study. If the patients agreed to participate, they were given a consent form to sign, after which they were asked to complete the survey in a private area to ensure confidentiality. Each participant was given ample time to complete the survey at their own pace.

**Sample Size Justification**

The sample size for this study was determined based on guidelines from previous studies on patient satisfaction and loyalty in healthcare settings. According to similar cross-sectional studies, a sample size of 300–400 participants is typically recommended to achieve sufficient statistical power for detecting medium-to-large effect sizes in multiple regression analyses [23,34,35]. Additionally, we considered the expected response rate and variability of the patient satisfaction and loyalty measures used in this context. Based on these considerations, a sample size of 350 participants was deemed appropriate for this study.

**Data Collection Tools**

Data were collected from patients attending public and private healthcare facilities by A.A.A. and M.A. These assistants were provided with standardized training on how to administer the Patient Satisfaction Questionnaire (PSQ-18) and the Patient Loyalty Questionnaire (PLQ) to ensure consistency and accuracy in data collection. The assessments were conducted with in-person interviews, depending on the patient’s preference and availability. All data collection procedures were supervised by the principal investigator to ensure adherence to the study protocol. These tools were chosen to ensure reliability, consistency, and comprehensiveness in capturing key factors influencing patient satisfaction and loyalty among Saudi patients attending healthcare facilities in the Riyadh region.

**1.**  **Demographic Questionnaire**

A brief demographic survey was used to gather background information on participants, including age, gender, educational level, the type of healthcare facility (public or private), and the frequency of healthcare visits. This data helped explore how these demographic factors influenced patient satisfaction and loyalty, enabling a more detailed analysis of correlations between patient characteristics and their experiences in healthcare settings.

**2.**  **Patient Satisfaction Questionnaire (PSQ-18)**

The **PSQ-18** is a widely used, validated instrument that measures patient satisfaction across several critical domains of healthcare delivery [36]. It is a shorter version of the original 50-item Patient Satisfaction Questionnaire (PSQ-III) and was chosen for its ease of use, brevity, and comprehensive coverage of essential aspects of patient satisfaction. The PSQ-18 has been validated in various healthcare contexts and has demonstrated high reliability and internal consistency, making it an appropriate tool for the Saudi healthcare setting.

The PSQ-18 assesses patient satisfaction across the following dimensions:

- **General Satisfaction:** Overall satisfaction with the healthcare services received.
- **Technical Quality:** Perception of the technical competence and expertise of healthcare providers, including the accuracy of diagnoses and appropriateness of treatments.
- **Interpersonal Manner:** Assessment of the healthcare provider’s communication skills, empathy, and respect for the patient’s needs.
- **Communication:** Clarity of explanations, the amount of information provided, and the healthcare provider’s ability to answer patient questions.
- **Financial Aspects:** Perceived fairness of costs and expenses related to the healthcare services.
- **Time Spent with the Doctor:** Perception of whether sufficient time was allocated to the patient during consultations.
- **Accessibility and Convenience:** Ease of scheduling appointments, waiting times, and availability of medical services when needed.

The questionnaire contains 18 items rated on a 5-point Likert scale ranging from 1 (strongly disagree) to 5 (strongly agree). Higher scores indicate higher levels of patient satisfaction. The PSQ-18 provides both an overall satisfaction score and scores for each specific domain, enabling the researchers to examine satisfaction levels across different aspects of care.

In this study, the PSQ-18 was translated into Arabic using a forward–backward translation method to ensure cultural relevance and comprehension. Bilingual experts who were familiar with both the medical context and Saudi culture performed the translation. The accuracy of the translation was verified by back-translation into English, followed by a pilot test with a small sample of Saudi patients to ensure clarity and appropriateness.

The validity of the Arabic version of the PSQ-18 was confirmed through this pilot testing, and adjustments were made based on feedback to improve clarity. The reliability of the scale in the Saudi context was assessed using Cronbach’s alpha, with the overall internal consistency for the PSQ-18 yielding a strong Cronbach’s alpha of 0.88, indicating high reliability.

**3.**  **Patient Loyalty Questionnaire (PLQ)**

To measure patient loyalty, a modified version of the Net Promoter Score (NPS) was used. The NPS is a widely accepted tool for assessing loyalty in various industries, including healthcare [37]. It measures the likelihood of patients recommending a healthcare facility to others and returning for future services. In this study, the Patient Loyalty Questionnaire (PLQ) was adapted from the NPS and consisted of the following two main components:

- Recommendation Likelihood: Participants were asked to rate, on a scale from 0 to 10, how likely they were to recommend the healthcare facility to family and friends. A higher score indicated a greater likelihood of recommendation, which is a strong indicator of loyalty and satisfaction with the services provided.
- Return Intent: Participants were asked to rate their intention to return to the same healthcare facility for future care. This item was also rated on a scale from 0 to 10, with higher scores indicating a stronger intent to continue using the same healthcare provider or facility.

The NPS system categorizes respondents into the following three groups based on their responses:

- Promoters (9–10): Patients who are highly loyal and likely to recommend the facility.
- Passives (7–8): Patients who are satisfied but not as enthusiastic or committed.
- Detractors (0–6): Patients who are unlikely to recommend the facility and may even share negative experiences.

By combining these two elements, the PLQ provided a comprehensive measure of patient loyalty, allowing the researchers to assess both the likelihood of patients recommending the facility and their intent to return for future care.

The Patient Loyalty Questionnaire (PLQ), adapted from the Net Promoter Score (NPS), assesses the following two main components: the likelihood of recommending the healthcare facility to others and the intent to return for future services.

Similar to the PSQ-18, the PLQ was translated into Arabic following the forward–backward translation process. This ensured that the scale was culturally and linguistically appropriate for the Saudi population. The translated version was pilot-tested for comprehensibility, and modifications were made accordingly.

Reliability for the Arabic version of the PLQ was also assessed using Cronbach’s alpha, which resulted in a value of 0.85, indicating good internal consistency and reliability of the scale within this study.

**Procedure**

The study was conducted between January and March 2024, following ethical approval from the King Saud University Institutional Review Board (IRB = 24–685). After obtaining approval, the researchers contacted the administrators of the selected healthcare facilities to seek permission for data collection. Once permission was granted, participants were approached in the outpatient waiting areas of the healthcare facilities.

Each participant was provided with a brief explanation of the study’s purpose, and informed consent was obtained before participation. Participants were assured that their responses would remain confidential and that they could withdraw from the study at any time without any consequences to their care. Data collection was conducted in a private area within the healthcare facility to ensure confidentiality and to minimize distractions. Each participant completed the standardized questionnaires (PSQ-18 and PLQ) and the demographic survey, which took approximately 15–20 min to complete.

**Data Analysis**

Data were analyzed using IBM SPSS Statistics software (Version 28). Descriptive statistics were calculated to summarize the demographic characteristics of the participants, including frequencies and percentages for categorical variables, as well as means and standard deviations for continuous variables.

For inferential analysis, Pearson correlation coefficients were used to assess the relationships between patient satisfaction (measured by the PSQ-18) and patient loyalty (measured by the PLQ). Pearson correlation was chosen to evaluate the strength and direction of linear associations between these continuous variables.

Multiple linear regression analyses were performed to identify the key predictors of patient satisfaction and loyalty. The independent variables included demographic factors (age, gender, and educational level) and healthcare-related factors (the type of facility, frequency of healthcare visits, and communication quality). These regression models were used to determine the extent to which each variable contributed to overall patient satisfaction and loyalty.

Before performing the regression analyses, key assumptions were tested, including linearity, homoscedasticity, multicollinearity, and independence of errors. The Durbin–Watson statistic was used to test for autocorrelation, and variance inflation factors (VIFs) were calculated to assess multicollinearity. All analyses were conducted with a significance level set at *p* < 0.05.

## 4. Results

The demographic characteristics of the 350 participants are summarized in Table 1. The sample had a fairly balanced gender distribution, with 54.6% male and 45.4% female participants. The majority of the participants were relatively young, with 61.4% aged between 18 and 39 years. Most participants held a university degree (64.3%), while 27.4% had completed secondary education, and only 8.3% had a primary education. In terms of healthcare facility usage, 52.6% of participants visited public healthcare facilities, while 47.4% used private healthcare services. Regarding the frequency of healthcare visits, 41.1% had visited healthcare facilities 2–3 times in the past year, followed by 30.0% who visited 4 or more times, and 28.9% who visited once.

Table 2 presents the patient satisfaction scores across different domains measured by the PSQ-18. Overall, patients reported high satisfaction levels, with technical quality receiving the highest mean score (4.2 ± 0.7), indicating positive perceptions of healthcare providers’ competence and performance. General satisfaction followed closely with a mean score of 4.1 ± 0.8. However, domains related to interpersonal interactions, such as interpersonal manner (4.0 ± 0.9) and communication (3.9 ± 0.8), scored slightly lower, suggesting areas for potential improvement in patient–provider relationships. Financial aspects had the lowest mean score (3.5 ± 1.0), reflecting concerns about the cost of care. The time spent with the doctor (3.8 ± 0.9) and accessibility and convenience (3.6 ± 1.1) were also relatively lower, indicating that patients may feel the need for better access to services and more personalized attention during consultations.

Table 3 presents the patient loyalty scores, measured by the following two key metrics: the recommendation likelihood and return intent. The mean score for the recommendation likelihood was 8.2 (SD = 1.1), indicating that most patients would recommend the healthcare facility to others, reflecting overall satisfaction and a positive healthcare experience. The mean score for the return intent was slightly lower at 8.0 (SD = 1.2), suggesting that, while patients are generally inclined to return to the same facility, there may be factors influencing their decision to seek care elsewhere.

In Table 4, Pearson correlation coefficients were calculated to assess the strength and direction of the linear associations between different domains of patient satisfaction (measured by the PSQ-18) and patient loyalty (measured by the PLQ). It is important to note that Pearson correlation only captures the linear relationship between variables and does not account for potential non-linear (e.g., polynomial) associations. As shown, general satisfaction exhibited the strongest positive linear correlation with patient loyalty (r = 0.65, *p* < 0.001), followed by communication (r = 0.62, *p* < 0.001) and interpersonal manner (r = 0.60, *p* < 0.001). Financial aspects had the weakest linear association with loyalty (r = 0.42, *p* < 0.001), suggesting that, while costs are a factor, they play a smaller role in predicting loyalty compared to other domains.

The results of the multiple linear regression analysis, presented in Table 5, reveal several significant predictors of patient satisfaction. Educational level and frequency of healthcare visits both showed strong associations with satisfaction, where patients with higher education levels (secondary: β = 0.18, *p* = 0.012; university: β = 0.23, *p* = 0.003) reported significantly higher satisfaction compared to those with primary education. Similarly, patients who visited healthcare facilities more frequently (2–3 times: β = 0.11, *p* = 0.025; 4 or more times: β = 0.17, *p* = 0.008) exhibited higher satisfaction scores. Additionally, being male (β = 0.12, *p* = 0.021) and attending a private healthcare facility (β = 0.15, *p* = 0.015) were also significant positive predictors of satisfaction. The model explains 68% of the variance in patient satisfaction, as indicated by the R^2^ value, with all significant predictors contributing meaningfully to the overall model (*p* < 0.001).

The results of the multiple linear regression analysis in Table 6 compare patient satisfaction and loyalty between public and private healthcare facilities. Private facilities consistently reported higher mean scores across all domains of satisfaction, including general satisfaction (mean = 4.3, SD = 0.7), technical quality (mean = 4.4, SD = 0.6), and communication (mean = 4.1, SD = 0.7). Public facilities reported lower mean scores, with general satisfaction at 3.9 (SD = 0.8) and communication at 3.7 (SD = 0.8). The differences between public and private facilities were statistically significant, with all *t*-values greater than −3.12 and *p*-values below 0.01, indicating that private facilities are perceived more positively by patients. Additionally, private facilities had a higher patient loyalty score (mean = 8.5, SD = 1.0) compared to public facilities (mean = 7.8, SD = 1.2), with a statistically significant difference (*t* = −5.35, *p* < 0.001).

The multiple regression analysis in Table 7 highlights the key predictors of patient loyalty. General satisfaction emerged as the strongest predictor, with a Beta coefficient of 0.48 (*p* < 0.001), indicating a significant and positive relationship between overall satisfaction and patient loyalty. Communication (β = 0.28, *p* < 0.001) and interpersonal manner (β = 0.22, *p* < 0.001) were also significant contributors, underscoring the importance of clear communication and positive patient–provider interactions in fostering loyalty. Additionally, financial aspects (β = 0.18, *p* = 0.008) and the time spent with the doctor (β = 0.15, *p* = 0.015) had smaller but still significant effects, suggesting that, while financial concerns and time with providers are important, their impact on loyalty is less than that of general satisfaction and communication.

The results summarized in Table 8 highlight the key factors influencing both patient satisfaction and loyalty. General satisfaction emerged as the strongest predictor for both outcomes, with a Beta coefficient of 0.48 for satisfaction and 0.55 for loyalty (*p* < 0.001), indicating that patients who are generally satisfied are more likely to remain loyal to their healthcare providers. Communication also had a significant impact, with Beta values of 0.35 for satisfaction and 0.42 for loyalty (*p* < 0.001), underscoring the importance of clear and effective communication in fostering both satisfaction and loyalty. Interpersonal manner was another critical factor, contributing significantly to both satisfaction (Beta = 0.22) and loyalty (Beta = 0.28), with *p*-values of less than 0.001. Other factors, such as the educational level (Beta = 0.18 for satisfaction and 0.15 for loyalty) and frequency of healthcare visits (Beta = 0.13 for satisfaction and 0.12 for loyalty), also showed positive associations, suggesting that higher education and more frequent interactions with healthcare services contribute to improved satisfaction and loyalty. The type of healthcare facility (private vs. public) had a significant effect, particularly on loyalty (Beta = 0.28, *p* < 0.001). In contrast, gender had a small but significant effect on satisfaction (Beta = 0.12, *p* = 0.021), but a non-significant effect on loyalty (Beta = 0.08, *p* = 0.110). Age was not significantly associated with either satisfaction or loyalty (*p* > 0.05).

## 5. Discussion

This study explores the multifaceted factors influencing patient satisfaction and loyalty among healthcare facilities in Riyadh, Saudi Arabia. The findings reveal that communication quality, general satisfaction, and interpersonal manner are critical determinants of both patient satisfaction and loyalty. In this expanded discussion, we will interpret these findings in relation to the existing literature and reflect on their implications for healthcare policy and practice in the Saudi context.

**Communication Quality as a Predictor of Satisfaction and Loyalty**

The central role of communication quality in shaping patient satisfaction and loyalty aligns with a wealth of literature in healthcare. Numerous studies have identified effective patient–provider communication as a core component of patient-centered care [38]. Almass et al. (2022), in their study of Saudi healthcare facilities, emphasized that clear, empathetic communication significantly enhances patient satisfaction. In the current study, communication quality emerged as one of the strongest predictors of both satisfaction and loyalty, reinforcing the notion that effective communication fosters trust and rapport, which are essential for long-term patient relationships [6].

Globally, patient-centered communication has been linked to improved clinical outcomes, higher adherence to treatment, and greater patient loyalty [39]. Effective communication not only ensures that patients understand their diagnosis and treatment plans but also enhances their overall healthcare experience, which, in turn, fosters loyalty to healthcare providers and institutions [40]. This finding is particularly important in the context of Saudi Arabia, where healthcare providers serve a culturally diverse population with varying expectations and communication needs. Ensuring that healthcare providers are trained in culturally sensitive communication techniques can help improve patient satisfaction and loyalty [41].

**General Satisfaction as a Key Driver of Loyalty**

General satisfaction, as measured by the PSQ-18, was the strongest predictor of patient loyalty. This finding is consistent with the global literature, which suggests that satisfied patients are more likely to continue using the same healthcare provider and recommend the facility to others [42,43]. In this study, general satisfaction encapsulated a variety of factors, including technical quality, interpersonal manner, and the overall healthcare environment. This multidimensional aspect of satisfaction mirrors the findings in studies from other healthcare systems, which have consistently shown that satisfied patients are more likely to exhibit loyalty behaviors, such as repeat visits and referrals [38,44,45].

In Saudi Arabia, patient satisfaction is not only a reflection of healthcare service quality but also a key performance indicator for healthcare organizations. With the Saudi Vision 2030 initiative aiming to enhance the quality of healthcare services, these findings underscore the importance of prioritizing patient satisfaction as a strategic goal for healthcare facilities [42]. As general satisfaction is closely tied to loyalty, healthcare institutions that invest in improving the patient experience will likely see long-term benefits, including higher patient retention rates and an enhanced reputation in the healthcare market [46].

**Interpersonal Manner and Patient Loyalty**

Interpersonal manner, defined as the empathy, respect, and attentiveness shown by healthcare providers, was another significant predictor of both patient satisfaction and loyalty. This finding is consistent with research conducted in various healthcare settings, which has demonstrated that patients value not only the technical competence of their healthcare providers but also the quality of the personal interactions they have with them [47]. The importance of interpersonal manner in healthcare reflects the broader shift towards patient-centered care, where the patient’s emotional and psychological needs are considered just as important as their physical health [48].

In the Saudi cultural context, interpersonal relationships are particularly important. The strong emphasis on family, respect, and social harmony in Saudi culture means that patients are likely to place a high value on the interpersonal aspects of their care. Healthcare providers who demonstrate empathy, active listening, and respect for patients’ cultural values are more likely to earn their trust and loyalty [49].

**Private vs. Public Healthcare Facilities: A Comparative Analysis**

One of the most striking findings of this study is the significant difference in patient satisfaction and loyalty between private and public healthcare facilities. Patients attending private healthcare facilities reported higher satisfaction and loyalty scores across all measured domains. This finding is consistent with research conducted in other Middle Eastern countries, including Qatar and the United Arab Emirates, where private healthcare providers have been found to offer superior service quality and a more patient-centered approach compared to public hospitals [23].

Several factors may contribute to the superior performance of private healthcare facilities in Saudi Arabia. Private institutions often have more resources to invest in infrastructure, technology, and staff training, which enables them to provide a more personalized and efficient service [14]. Moreover, the competitive nature of the private healthcare market incentivizes these facilities to prioritize patient satisfaction as a means of attracting and retaining patients. In contrast, public healthcare facilities, which often operate under resource constraints and serve a larger volume of patients, may struggle to provide the same level of personalized care [50].

Policymakers should consider implementing initiatives to enhance the quality of care in public facilities, such as increasing investment in staff training, reducing patient-to-provider ratios, and adopting best practices from the private sector [5].

**Educational Level and Healthcare Experience**

Educational level emerged as a significant predictor of patient satisfaction and loyalty, with patients who had higher levels of education reporting greater satisfaction. This finding is consistent with previous research, which has suggested that more educated patients tend to have higher expectations of healthcare services and are more critical in their evaluations [51]. Educated patients are often better equipped to navigate the healthcare system, ask informed questions, and advocate for their healthcare needs, which may contribute to their higher levels of satisfaction [52].

Where the healthcare system is undergoing rapid transformation, the growing number of educated patients presents both opportunities and challenges for healthcare providers [13]. On one hand, educated patients may push healthcare providers to improve service quality and offer more patient-centered care [2]. On the other hand, healthcare providers must be prepared to meet the higher expectations of these patients, who may demand more information, better communication, and more personalized care [53].

To address these challenges, healthcare providers should consider adopting more tailored approaches to patient care, particularly for patients with higher levels of education. For example, providing more detailed explanations of medical procedures and treatment options, as well as involving patients more actively in decision making, can help meet the expectations of educated patients and improve their satisfaction with healthcare services.

**Frequency of Healthcare Visits and Patient Loyalty**

The frequency of healthcare visits was another important predictor of patient satisfaction and loyalty. Patients who visited healthcare facilities more frequently reported higher satisfaction and loyalty levels. This finding aligns with existing research, which suggests that frequent interactions with healthcare providers can help build trust, strengthen the patient–provider relationship, and foster loyalty [54].

In Saudi Arabia, frequent visits to healthcare facilities may be particularly important for patients with chronic conditions, such as diabetes and hypertension, who require ongoing care and follow-up [55]. These patients are likely to develop long-term relationships with their healthcare providers, which can enhance their satisfaction and loyalty. However, the finding also highlights the need for healthcare providers to ensure that each interaction with the patient is meaningful, regardless of how often they visit [56]. Even routine visits should be seen as an opportunity to reinforce trust, provide personalized care, and enhance the overall patient experience [57].

**Cultural Context and Healthcare Expectations**

The cultural context of Saudi Arabia plays a crucial role in shaping patients’ expectations and experiences of healthcare. Saudi patients place a strong emphasis on privacy, respect, and modesty, particularly in relation to gender-segregated care [58]. This cultural emphasis may influence patients’ expectations regarding communication, interpersonal manner, and the availability of female healthcare providers for female patients [59].

While this study did not directly measure the influence of cultural factors on patient satisfaction and loyalty, the findings suggest that healthcare providers who are attuned to the cultural needs of their patients are more likely to foster satisfaction and loyalty [16]. For instance, providing gender-appropriate care and ensuring that patients’ religious and cultural preferences are respected can help healthcare providers build stronger relationships with their patients [60].

## 6. Limitations of the Study

While this study offers valuable insights into the predictors of patient satisfaction and loyalty, there are several limitations that should be considered. First, the wide age range of participants, which spanned from 18 to 75 years, may have introduced variability in the findings. Younger patients often have different healthcare needs, expectations, and levels of healthcare engagement compared to older patients. This age variation could have affected the satisfaction and loyalty scores, as older patients might prioritize different aspects of care (e.g., accessibility and communication) than younger patients. Future studies should consider stratifying the sample by age to explore how these factors may differ across age groups.

In addition, the study did not account for the characteristics of different disease factors. Patients with chronic conditions, such as diabetes or cardiovascular diseases, may have different experiences and expectations compared to those with acute or less complex conditions. The severity and type of disease could significantly influence patient satisfaction and loyalty, as chronic patients often have more frequent interactions with healthcare providers and may prioritize continuity of care. Incorporating disease severity and type into future analyses could provide a more comprehensive understanding of how these factors affect patient outcomes.

Finally, the study included participants from various healthcare units, ranging from primary care to specialty clinics, which may have introduced variability in patient satisfaction and loyalty. Different units provide varying levels of care, and patients may have different expectations depending on the type of care they receive. For instance, patients attending specialty clinics might have different satisfaction drivers than those seeking routine primary care. Future research should focus on specific healthcare units to control for this variability and offer a clearer picture of satisfaction and loyalty within different care settings. Also, the potential influence of cultural, religious, and organizational factors, as discussed in the Introduction, were not directly measured in the study.

## 7. Conclusions

This study provides valuable insights into the factors influencing patient satisfaction and loyalty among Saudi patients attending healthcare facilities in the Riyadh region. The findings highlight the critical role of general satisfaction, communication, and interpersonal manner in fostering patient loyalty, with patients from private healthcare facilities reporting higher satisfaction and loyalty levels compared to those from public facilities. Educational level and the frequency of healthcare visits also emerged as significant predictors, suggesting that more frequent interactions with healthcare providers contribute to stronger patient–provider relationships. These results emphasize the need for healthcare providers and policymakers to prioritize improving communication, interpersonal skills, and overall care quality to enhance patient experiences and foster long-term loyalty. Tailoring services to meet the diverse needs of the patient population, particularly in terms of educational background and visit frequency, will be essential in achieving these goals.

## Figures and Tables

**Table 1:** Demographic characteristics of participants (*n* = 350).

Characteristic	Frequency (n)	Percentage (%)
Age (years)		
18–29	96	27.4
30–39	119	34.0
40–49	82	23.4
50 and above	53	15.2
Gender		
Male	191	54.6
Female	159	45.4
Educational Level		
Primary	29	8.3
Secondary	96	27.4
University Degree	225	64.3
Type of Healthcare Facility		
Public	184	52.6
Private	166	47.4
Frequency of Healthcare Visits		
Once	101	28.9
2–3 times	144	41.1
4 or more times	105	30.0

**Table 2:** Patient satisfaction scores across PSQ-18 domains (n = 350).

PSQ-18 Domain	Mean Score	Standard Deviation (SD)
General Satisfaction	4.1	0.8
Technical Quality	4.2	0.7
Interpersonal Manner	4.0	0.9
Communication	3.9	0.8
Financial Aspects	3.5	1.0
Time Spent with Doctor	3.8	0.9
Accessibility and Convenience	3.6	1.1

**Table 3:** Patient loyalty scores (n = 350).

Loyalty Metric	Mean Score	Standard Deviation (SD)
Recommendation Likelihood	8.2	1.1
Return Intent	8.0	1.2

**Table 4:** Correlation between patient satisfaction and loyalty scores (n = 350).

Variable	Patient Loyalty	*p*-Value
General Satisfaction	0.65	<0.001
Technical Quality	0.58	<0.001
Interpersonal Manner	0.60	<0.001
Communication	0.62	<0.001
Financial Aspects	0.42	<0.001
Time Spent with Doctor	0.45	<0.001
Accessibility and Convenience	0.38	<0.001

0.00–0.30: weak correlation. 0.30–0.50: moderate correlation. 0.50–1.00: strong correlation.

**Table 5:** Multiple regression analysis predicting patient satisfaction (n = 350).

Predictor Variable	Beta Coefficient	Standard Error	*p*-Value
Gender (Male)	0.12	0.05	0.021
Age	−0.08	0.04	0.084
Educational Level (Reference: Primary)			
Secondary	0.18	0.07	0.012
University	0.23	0.08	0.003
Type of Healthcare Facility (Private vs. Public)	0.15	0.06	0.015
Frequency of Healthcare Visits (Reference: Once)			
2–3 times	0.11	0.04	0.025
4 or more times	0.17	0.05	0.008

**Table 6:** Comparison of patient satisfaction and loyalty between public and private healthcare facilities (n = 350).

Variable	Public Facility (Mean ± SD)	Private Facility (Mean ± SD)	*t*-Value	*p*-Value
General Satisfaction	3.9 ± 0.8	4.3 ± 0.7	−4.22	<0.001
Technical Quality	4.0 ± 0.7	4.4 ± 0.6	−4.05	<0.001
Interpersonal Manner	3.8 ± 0.9	4.2 ± 0.8	−3.90	<0.001
Communication	3.7 ± 0.8	4.1 ± 0.7	−4.00	<0.001
Financial Aspects	3.3 ± 1.0	3.7 ± 0.9	−3.12	0.002
Loyalty Score	7.8 ± 1.2	8.5 ± 1.0	−5.35	<0.001

**Table 7:** Predictors of patient loyalty: multiple regression analysis (n = 350).

Predictor Variable	Beta Coefficient	Standard Error	*p*-Value
General Satisfaction	0.48	0.06	<0.001
Interpersonal Manner	0.22	0.05	<0.001
Communication	0.28	0.04	<0.001
Financial Aspects	0.18	0.06	0.008
Time Spent with Doctor	0.15	0.05	0.015

**Table 8:** Summary of key factors influencing patient satisfaction and loyalty (n = 350).

Factor	Beta Coefficient (Patient Satisfaction)	*p*-Value (Patient Satisfaction)	Beta Coefficient (Patient Loyalty)	*p*-Value (Patient Loyalty)
Gender (Male)	0.12	0.021	0.08	0.110
Age	−0.08	0.084	−0.06	0.142
Educational Level (Reference: Primary)				
-Secondary	0.18	0.004	0.15	0.013
-University Degree	0.23	0.003	0.20	0.010
Type of Healthcare Facility (Private vs. Public)	0.15	0.015	0.28	<0.001
Frequency of Healthcare Visits (Reference: Once)				
-2–3 times	0.11	0.025	0.10	0.030
-4 or more times	0.17	0.008	0.12	0.019
General Satisfaction	0.48	<0.001	0.55	<0.001
Interpersonal Manner	0.22	<0.001	0.28	<0.001
Communication	0.35	<0.001	0.42	<0.001

## Data Availability

All data are available within the manuscript.
